# Does mammographic density predict survival in women with invasive breast cancer? The need to account for potential confounding from cancer stage and overdiagnosis

**DOI:** 10.1016/j.breast.2023.07.004

**Published:** 2023-07-10

**Authors:** Katy JL. Bell, Meagan Brennan

**Affiliations:** Sydney School of Public Health, Faculty of Medicine and Health, The University of Sydney, Sydney, 2006, Australia; Westmead Clinical School, Faculty of Medicine and Health, The University of Sydney, Sydney, 2006, Australia

## Abstract

• The negative findings from the linked study suggest breast density may not affect prognosis after a breast cancer diagnosis.
• Cancer stage and overdiagnosis are potential confounders of the relationship between breast density and health outcomes.
• The prognostic value of changes in mammographic density over time may be explored in future research.

High breast density on mammography is a well-established risk factor for a breast cancer diagnosis [1,2]. There is interest in whether breast density might also predict how aggressive a breast cancer is, including the likelihood of causing death. If mammographic features at the time of cancer diagnosis were found to have prognostic value, and this added to the prognostic value of histopathology, staging, and other clinical features, then this information could have clinical utility for management decisions. For example, choice of treatment sequencing could be impacted, such as the decision to use neoadjuvant chemotherapy.

In this issue of The Breast, Dr Sturesdotter and colleagues investigate the first part of this question (do mammographic features have prognostic value) in over 1100 women diagnosed with invasive cancer who were participating in the Malmö Diet and Cancer study [3]. Using this large population-based sample of women, they present a robust evaluation of potential associations between mammographic findings at the time of the cancer diagnosis and subsequent risk of dying from breast cancer, including separate analysis for screen-detected breast cancer and clinically detected cancer (stratified analysis) and adjustment for many potential confounders. Unfortunately, data were not available for cancer stage which is an important potential confounder that is known to be associated with mammographic density (cancers in dense breasts tending to be diagnosed at a later stage) and with breast cancer mortality.

Lack of adjustment for stage and other unmeasured confounders may bias estimates of association between mammographic appearances and long-term prognosis. Figure 1 explores possible effects that unadjusted confounders may have for the case of screen detected cancers (Fig. 1). Unmeasured confounders might explain the authors’ finding of a possible increased mortality risk for women with high density where the breast cancer was screen-detected (HR 1.45, CI 0.87 to 2.43). In this scenario, high density at time of cancer diagnosis may be acting as a marker for higher stage cancer, through its association with high density on previous screening mammography and potential masking of early stage cancer [4]. Early-stage screen detected cancers are also liable to overdiagnosis – lesions that are invasive cancer on histopathology (not a false positive) but would never have caused symptoms or death if left undetected and untreated [[5], [6], [7], [8]]. Women with low density on screening mammography may be more likely to have an overdiagnosed cancer, although this has not yet been demonstrated empirically. This uneven distribution of overdiagnosed cancers may also cause an apparent increased mortality risk for high density. Both potential confounders may be less prominent in the setting of clinically detected cancers. Furthermore, as they would both tend to bias in favour of finding an effect, this adds confidence that the finding of no increased mortality risk in the clinically detected cancer group may be real.

**Fig. 1:**
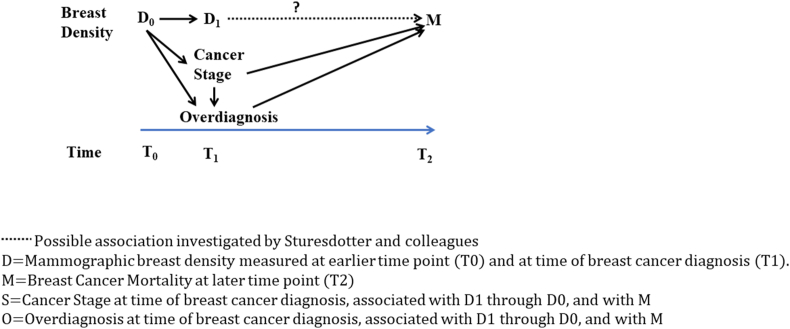
Potential confounders of association between mammographic breast density at time of screen detected invasive cancer diagnosis and breast cancer mortality.

The method of measurement of breast density on mammography may also be important. Density measured using the “qualitative” 4 category Breast Imaging Reporting and Data System (BI-RADS, the method used in the current study) has been found to predict risk of breast cancer diagnosis, with both as a single density measurement [1,2], and changes in density measurements over time found to be predictive [9,10]. However, there is considerable inter-rater and intra-rater variability in this method of determining breast density. The current Malmö study defined ‘dense breast tissue’ as BIRADS 4 and compared their outcomes to ‘fat involuted’ and ‘moderately dense’ BIRADS 1–3 combined. Other studies on breast density have tended to define ‘dense breast tissue’ as BIRADS 3–4, with comparison to BIRADS 1–2. Consistency in definitions is important to allow comparison of results between studies.

With the transition to digital mammography [11], quantitative measurement of volumetric mammographic breast density is possible, which may decrease measurement variability in density estimates, especially if repeated measures using the same technology are available. A recent population-based study of screening mammography using repeated measures of volumetric breast density found that density tended to decrease over time whether or not a woman was subsequently diagnosed with cancer [10]. However, this decrease was significantly attenuated in the breast ipsilateral to that where a cancer was subsequently detected (in women with a breast cancer diagnosis) compared to the breasts of control women (who did not develop cancer), providing incremental prediction to baseline volumetric measurement alone [10]. It is possible that breast density measured in this way could also provide incremental prognostic information for women diagnosed with breast cancer.

The overall negative findings from the current Malmo study offer some reassurance that while dense breast tissue is a risk factor for breast cancer, it may not affect prognosis once cancer is diagnosed. However, this should be interpreted with caution due to potential confounders and the possibility that breast-specific mammographic density changes over time may provide prognostic information on health outcomes beyond the cancer diagnosis. Future research seeking to investigate that hypothesis should include consideration of ways to account for potential confounders including cancer stage and overdiagnosis.

## Funding statement

KB is the recipient of an 10.13039/501100000925NHMRC Investigator Grant (#1174523). The funder played no role in the study design, in the collection, analysis and interpretation of data; in the writing of the manuscript; and in the decision to submit the manuscript for publication.

## Declaration of competing interest

None declared.
